# Association of rs4618210A>G variant in *PLCL2* gene with myocardial infarction: A case-control study in Iran

**DOI:** 10.34172/jcvtr.2020.49

**Published:** 2020-11-28

**Authors:** Najmeh Ramezanpour, Mahboobeh Nasiri, Omid Reza Akbarpour

**Affiliations:** ^1^Department of Biology, Islamic Azad University, Arsanjan Branch, Arsanjan, Iran

**Keywords:** Polymorphism, PLCL2, Myocardial Infraction

## Abstract

***Introduction:*** Myocardial infarction (MI) is the leading cause of death all over the world. The pivotal roles of Phospholipase C like 2 gene (*PLCL2*) in calcium homeostasis and immune responses make this gene as a potential candidate for its role in MI pathogenesis. The present study was undertaken to investigate whether rs4618210A>G polymorphism in *PLCL2* gene contribute to MI etiology.

***Methods:*** A hospital-based case-control study with 600 subjects, including 300 MI patients and 300controls, was conducted. Genotyping of *PLCL2* rs4618210 polymorphism was performed using amplification refractory mutation system-polymerase chain reaction (ARMS PCR) method. Data were analyzed using logistic regression analysis.

***Results:*** No significant association was found between the *PLCL2* rs4618210 alleles and MI risk.However, a significantly increased risk of MI was observed among carriers of the AG genotype (OR= 1.91; 95% CI = 1.24 - 2.93; *P *= 0.003) compared with AA homozygote. In a dominant mode of inheritance for G allele (GG + AG vs. AA), the frequency of the carriers of at least one G allele was higher in cases compared to controls (OR= 1.56; 95% CI: 1.03 – 2.36; *P *= 0.037).

***Conclusion:*** Our study provided further evidence that *PLCL2* gene polymorphism may serve as a prognostic marker for MI.

## Introduction

The influence of heritable materials in the pathogenesis of the myocardial infarction (MI) has been proved in many independent studies.^1-3^ Recently, genome-wide association studies (GWAS) improved the way of exploring the susceptible loci and genetic polymorphisms contributing to the pathogenesis of MI.^4^ Performing a large-scale GWAS in the Japanese population, Hirokawa et al introduced *PLCL2* and *AP3D1-DOT1L-SF3A2* as the two novel candidate genes for MI.^5^ Phospholipase C like 2 gene (*PLCL2*, OMIM#614276) located on the short arm of human chromosome 3 at 3p24.3 cytogenetic band and the coding information of the gene recorded in 12 exons.^6^ *PLCL2*is abundantly expressed in skeletal muscles, but also detectable in lymphocytes and platelets, Brain, liver, thymus, and kidney.^6^ The catalytically inactive PLCL2 enzyme binds to inositol 1,4,5-trisphosphate [ins(1,4,5)P3] via the receptors existing on endoplasmic reticulum (ER) membrane and allows calcium release by forming vesicles. Alterations in PLCL2 can interfere with normal calcium homeostasis which results in abnormal proliferation, migration, and contraction of vascular smooth muscle cells (VSMCs) as the main events leading to atherosclerosis.^7-9^ Furthermore, the tissue distribution of the PLCL2 (e.g. lymphocytes and platelets; crucial components of atherosclerosis) may reflect the participation of this gene in atherosclerosis complex process through inflammation and immune responses.^9^

The unique features of the PLCL2 in calcium signaling pathway and subsequently in atherosclerosis led us to speculate that it may associate with myocardial infarction. To investigate this association, we compared the genotype distribution of the rs4618210A>G intron variant between two groups of MI patients and healthy controls.

## Materials and Methods

### Study population

This hospital-based case-control study composed of 300 cases with MI and 300 healthy volunteers as a control group. MI patients were enrolled among all the eligible subjects who admitted by chest pain in the Heart Clinic of the Vali-e-Asr hospital, Fasa, Iran during a period of six months prior to blood sampling between March and August 2016. Control subjects were at the same age and sex as the cases. Control subjects declared that they did not have a previous sign of heart problem or MI. MI patients without any age limitation and both genders were recruited among those with positive result of the troponin measurement. Patients with Diabetes mellitus, hypertension, smoking, and family history of heart diseases were included in the study to evaluate the association of these conditions with the risk of MI. Systolic blood pressure higher than 140 mmHg or diastolic blood pressure higher than 90 mmHg and fasting blood glucose level >126 mg/dl or dependency to insulin or other hypoglycemic drugs were considered as hypertension and diabetes mellitus respectively. Family history only checked among first and second relatives. Smoking status was defined as current smokers (positive group) and never smokers (negative group). Regarding the families with more than one affected patients with MI, only one patient was included. Patients who had recently performed heart surgery as well as patients with more than one MI were excluded from the study. Information about the lipid profile (LDL, HDL, and TG) of all subjects in both case and control groups was documented by checking the laboratory test result. Demographic characteristics including age, gender, weight, and height were asked at the time of sampling.

### Molecular analysis

**Figure 1:**
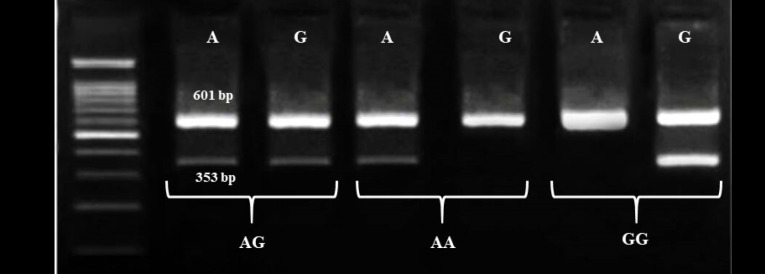
Genotyping of the *PLCL2* rs4618210 polymorphism using ARMS PCR

Following DNA extraction using standard salting out method, amplification refractory mutation system -polymerase chain reaction (ARMS PCR) method was used to amplify the genomic region containing the rs4618210A>G variant and its flanking sequences. The PCR mix prepared following the Yekta Tajhiz Azma kit instruction. The primers for ARMS PCR designed using primer 1 software after some modifications as

follows (polymorphic alleles are bold and underlined); forward outer (FO): 5’ GAGTCCTTTGTGTCTCGCCTTG 3’; reverse outer (RO): 5’ CTTCCCTGCCTTGCTTTTTCCATA 3’; reverse inner (RI) for A-allele: 5’ CCCTAAACAATGAGTTTTGTCTTTTTAT**T** 3’; and RI for G-allele: 5’ CCCTAAACAATGAGTTTTGTCTTTTTAT**C** 3’. The amplification reaction was done in the thermal condition including pre-denaturation at 95°C for 5 minutes , 30 cycles of consecutive steps of denaturation at 95°C for 45 sec, annealing at 61°C for 45 seconds and extension at 72°C for 45 seconds, and the final extension for 5 minutes at 72°C. The specified interaction of primers resulted in the non-allele specific product with 601 bp in length and the allele-specific product of 353 bp on 2.5% agarose gel (Figure 1).

### Statistical analysis

SPSS version 19 applied to statistically analyze the data. Quantity variables were presented as mean ± standard deviation (SD) and the differences between case and control groups was assessed using *t* test. Frequency and percent were used to represent categorical variables. The accordance of the observed genotype frequencies with Hardy-Weinberg equilibrium was tested using the Chi-square test. The association between *PLCL2*rs4618210 genotypes and MI was examined accounting the odds ratio (OR) and confidence interval (95%CI) in a monovariate logistic regression model. Logistic regression also used to explore the probable influence of some risk factors on the risk of MI. *P* values of less than 0.05 considered statistically significant in all analyses.

## Results

### Baseline characteristics of the study subjects

The mean age of the MI cases (63.20 ± 11.07 year) revealed no significant difference to the mean age of the controls (62.25 ± 12.06 year) with the *P* value of 0.32. The male to female ratio was 1.46 in patient group. Most of the MI cases were in the age above 50 years. The mean of the body mass index (BMI), Low-density lipoprotein (LDL), high-density lipoprotein (HDL) and triglyceride (TG) was significantly higher in cases compared to controls (all *P* values ≤ 0.001) (Table 1).

**Table 1:** Demographic and anthropometric characteristics and lipid profile of cases and controls

**Variable**	**Cases**	**Controls**	***P*** ^a^
Total	300	300	-
Age (Mean ± SD)	63.20 ± 11.07	62.25 ± 12.06	0.32
Age at diagnosis (Mean ± SD)	61.13 ± 9.46	-	-
Sex ratio (male: female)	178:122	174:126	0.74
Weight	71.80 ± 4.46	69.46 ± 8.00	≤ 0.001*
BMI, kg/m^2^	42.36 ± 1.99	40.49 ± 3.34	≤ 0.001*
LDL, mg/dL	86.73 ± 11.38	76.45 ± 11.42	≤ 0.001*
HDL, mg/dL	41.79 ± 6.32	39.14 ± 10.73	≤ 0.001*
TG, mg/dL	179.05 ± 38.00	139.59 ± 33.34	≤ 0.001*

Abbreviations: SD, standard deviation ^a^Student *t* test *Statistically significant.

### Association analysis

Allele frequencies and genotype distributions in cases and controls are shown in Table 2. Deviation from Hardy-Weinberg equilibrium was not seen in the control group (χ^2^ = 0.89, df = 1, *P* = 0.34). The frequency of the AG genotype was significantly higher in cases compared to controls (70% vs. 52.7%) and resulted in an increased risk of MI (OR: 1.91; 95%CI: 1.24 – 2.93; *P* = 0.003). Carriers of at least one G allele (GG + AG vs. AA) had a higher risk of MI (OR: 1.56; 95%CI: 1.03 – 2.36; *P* = 0.037). The frequency of the G allele in control and case groups was 51.65% and 49.7% respectively. The difference between the frequencies of the G allele between groups did not reach statistical difference (OR: 0.92; 95%CI: 0.73 – 1.16; *P* = 0.488).

**Table 2:** Alleles and genotypes distributions of the *PLCL2* rs4618210 polymorphism in cases and controls

**SNP**	**Variables**	**Cases n (%)**	**Controls n (%)**	***P*** ^a^	**OR (95% CI)**
rs4618210	Genotypes				
	AA	46 (15.3)	66 (22)	-	Reference
	AG	210 (70)	158 (52.7)	0.003*	1.91 (1.24 – 2.93)
	GG	44 (14.7)	76 (25.3)	0.492	0.83 (0.49 – 1.41)
	GG+AG	254 (84.7)	234 (78)	0.037*	1.56 (1.03 - 2.36)
	Alleles				
	A	302 (50.3)	290 (48.35)	-	Reference
	G	298 (49.7)	310 (51.65)	0.488	0.92 (0.73 - 1.16)

^a^Logistic regression analysis *Statistically significant

The frequency of hypertension in MI groups was significantly higher than controls and the data support the increased in the risk of MI among patients with hypertension (*P* ≤ 0.001). Diabetes mellitus, family history for heart diseases and smoking were also risk factor for MI (*P* ≤ 0.001) (Table 3).

**Table 3:** Association of the risk factors with MI

**Risk factor**	**Condition**	**MI** **Number (%)**	**Controls** **Number (%)**	***P*** **value** ^a^
Hypertension	No	6 (2)	297 (99)	≤ 0.001*
	Yes	294 (98)	3 (1)	
Diabetes	No	138 (46)	298 (99.3)	≤ 0.001*
	Yes	16 (54)	2 (0.7)	
Smoking	No	133 (44.3)	298 (99.3)	≤ 0.001*
	Yes	167 (55.7)	2 (0.7)	
Family history	No	3 (1)	291 (97)	≤ 0.001*
	Yes	297 (99)	9 (3)	

^a^Logistic regression analysis *Statistically significant.

## Discussion

Most of the predisposing loci known for their association with MI contained the genes of the immune system, inflammation and lipid metabolism.^2,11-13^ Hirokawa et al^5^ in their large GWAS found two novel genes for their association with MI. A strong association with the protective influence of the minor G-allele of the *PLCL2*rs4618210A>G intronic variant and the occurrence of MI was found in the Japanese population (*P* = 2.60×10-^9^; OR: 0.91). Qian et al^14^ investigated the possible influence of the *PLCL2* rs4618210 polymorphism in the pathogenesis of CAD in the Chinese population. They showed a higher frequency for G allele among cases with CAD compared to controls, with more than one-fold greater risk of CAD among carriers of G-allele. The distribution of the genotypes showed no significant difference between cases with CAD and controls, while the genotype frequencies significantly differed in the CAD patients with MI compared to controls (*P* < 0.05).

The results of our study focusing on the association of the mentioned SNP with MI was consistent with the pattern of relationship reported by Hirokawa et al However, no significant difference was found between the frequencies of the alleles between groups. In our present study, the frequency of the minor G-allele in cases with MI was higher than the alternate frequency in Japan but lower than the estimated frequency in China. While the frequency of this allele in controls was higher than the reported frequencies in both populations. The discrepancies may result from the inter-population heterogeneity, ethnic dependency and partly by anthropometric characteristics of the recruited individuals.

MI is a complex heterogeneous disease and making a connection between the mechanisms in which the *PLCL2* gene contribute in the pathogenesis of MI seems absolutely difficult. To achieve the best logical explanation for this association, we flashback to some previous reports investigating the role of *PLCL2* gene in the pathobiology of human diseases. In a GWAS by Mells et al an association between *PLCL2* rs1372072 polymorphism and risk of Primary biliary cirrhosis was found.^15^ In the other study, Sawcer et al investigated the cell-mediated immune mechanism in multiple sclerosis and reported an increased risk of disease in carriers of *PLCL2* rs9821630A>G polymorphism.^16^ An increase in the risk of rheumatoid arthritis was shown for *PLCL2* rs4535211 polymorphism.^17^ In recent studies, Arismendi et al and Tsoi et al found an association between *PLCL2*rs1372072 and systemic sclerosis,^18^ and *PLCL2*rs4685408 and Psoriasis^19^ respectively. All the above-mentioned disorders passing through generations as multifactorial complex diseases and considered as autoimmune diseases. Then, it is probable that *PLCL2* gene plays its role as a promoter of the immune reactions leading to atherosclerosis.

## Conclusion

Our findings implied that rs4618210 might contribute to the etiology of MI, but further investigations in other population are inevitable to make a valid conclusion. Further investigation considering the association between *PLCL2*rs4618210 polymorphism and the severity of the disease is suggested which our study limitation is.

## Acknowledgements

The authors present their sincere thanks to all the participants and the staffs of the Vali-e-Asr Hospital, Fasa, Iran for their kind collaboration in blood sampling.

## Competing interest

None.

## Ethical approval

The study was approved by the local committee in our department at Islamic Azad University, Arsanjan Branch.(16030503942005) All subjects signed the written informed consent form prior to blood sampling. The study was approved by the local committee in our department.

## Funding

The study was financially supported by Islamic Azad University, Arsanjan, Iran.
